# Retrospective Cohort Study of Additional Procedures and Transplant‐Free Survival for Patients With Functionally Single Ventricle Disease Undergoing Staged Palliation in England and Wales

**DOI:** 10.1161/JAHA.123.033068

**Published:** 2024-07-03

**Authors:** Qi Huang, Deborah Ridout, Victor Tsang, Nigel E. Drury, Timothy J. Jones, Hannah Bellsham‐Revell, Elena Hadjicosta, Anna N. Seale, Chetan Mehta, Christina Pagel, Sonya Crowe, Ferran Espuny‐Pujol, Rodney C. G. Franklin, Katherine L. Brown

**Affiliations:** ^1^ Clinical Operational Research Unit University College London London United Kingdom; ^2^ Population, Policy and Practice Programme Great Ormond Street Institute of Child Health, University College London London United Kingdom; ^3^ Great Ormond Street Hospital Biomedical Research Centre London United Kingdom; ^4^ Institute of Cardiovascular Science University College London London United Kingdom; ^5^ Paediatric Cardiology and Cardiac Surgery Birmingham Children’s Hospital Birmingham United Kingdom; ^6^ Institute of Cardiovascular Sciences University of Birmingham Birmingham United Kingdom; ^7^ Paediatric Cardiology Evelina London Children’s Hospital London United Kingdom; ^8^ Paediatric Cardiology Royal Brompton and Harefield NHS Foundation Trust London United Kingdom

**Keywords:** congenital heart disease, Fontan surgery, mortality, reintervention, univentricular heart, Congenital Heart Disease, Cardiovascular Surgery

## Abstract

**Background:**

Reinterventions may influence the outcomes of children with functionally single‐ventricle (f‐SV) congenital heart disease.

**Methods and Results:**

We undertook a retrospective cohort study of children starting treatment for f‐SV between 2000 and 2018 in England, using the national procedure registry. Patients were categorized based on whether they survived free of transplant beyond 1 year of age. Among patients who had transplant‐free survival beyond 1 year of age, we explored the relationship between reinterventions in infancy and the outcomes of survival and Fontan completion, adjusting for complexity. Of 3307 patients with f‐SV, 909 (27.5%), had no follow‐up beyond 1 year of age, among whom 323 (35.3%) had ≥1 reinterventions in infancy. A total of 2398 (72.5%) patients with f‐SV had transplant‐free survival beyond 1 year of age, among whom 756 (31.5%) had ≥1 reinterventions in infancy. The 5‐year transplant‐free survival and cumulative incidence of Fontan, among those who survived infancy, were 93.4% (95% CI, 92.4%–94.4%) and 79.3% (95% CI, 77.4%–81.2%), respectively. Both survival and Fontan completion were similar for those with a single reintervention and those who had no reinterventions. Patients who had >1 additional surgery (adjusted hazard ratio, 3.93 [95% CI, 1.87–8.27] *P*<0.001) had higher adjusted risk of mortality. Patients who had >1 additional interventional catheter (adjusted subdistribution hazard ratio, 0.71 [95% CI, 0.52–0.96] *P*=0.03) had a lower likelihood of achieving Fontan.

**Conclusions:**

Among children with f‐SV, the occurrence of >1 reintervention in the first year of life, especially surgical reinterventions, was associated with poorer prognosis later in childhood.

**Nonstandard Abbreviations and Acronyms**
aHR: adjusted hazard ratio
ANZFR: Australia and New Zealand Fontan Registry
aSHR: adjusted subdistribution hazard ratio
f‐SV: functionally single‐ventricle
HLHS: hypoplastic left heart syndrome
NCHDA: National Congenital Heart Diseases Audit
ONS: Office of National Statistics
SVR: Single Ventricle Reconstruction

**Clinical Perspective**

**What Is New?**

- A population‐based analysis of all 3307 children born between 2000 and 2018 in England and Wales and palliated for functionally single‐ventricle disease found that 32.6% required ≥1 additional procedures during infancy, with multiple additional procedures in 11.3%.
- Among 909 children who either died (755), underwent transplant (11), or were censored as <1 year old at data harvest (143), 235 (25.8%) had an additional surgery in infancy, compared with 399 (16.6%) among 2398 children who had survived beyond 1 year old (*P*<0.001).
- In 2398 children who survived beyond 1 year old, multiple surgeries in infancy were strongly linked to risk of death (adjusted hazard ratio, 3.93 [95% CI, 1.87–8.27] *P*<0.001) and multiple catheter reinterventions in infancy were linked to a lower likelihood of achieving Fontan (adjusted subdistribution hazard ratio, 0.71 [95% CI, 0.52–0.96] *P*=0.03).

**What Are the Clinical Implications?**

- Clinicians and families affected by functionally single‐ventricle disease should be prepared for reinterventions, since they are common, and when multiple reinterventions are required in infancy, this is associated with poorer outcome.
- Complex features in functionally single‐ventricle disease are linked to the occurrence of reinterventions; the combination of complexity and multiple reinterventions, especially surgery, is linked to poorer prognosis even among those who survive the first year of life.

1

Functionally single‐ventricle (f‐SV) disease represents the most complex group of congenital heart conditions. Given recent evolution in the management of these conditions with a historically high mortality, population‐based reports reflecting the outcomes of contemporary treatment pathways are sparse.^1^ Recognizing this knowledge gap, we recently reported long‐term survival rates among a population‐based cohort of patients born and treated for f‐SV in England and Wales since the year 2000: the survival rates at 1 year and 5 years were 76.8% (95% CI, 75.3%–78.2%) and 72.1% (95% CI, 70.6%–73.7%), respectively.^2^ Given the anatomic complexity and the need for a series of palliative surgeries in children with f‐SV,^3^ additional interventions occurring separately to staged palliation, are frequently needed.^4^, ^5^, ^6^, ^7^ We studied reinterventions in the f‐SV population born in England and Wales between 2000 and 2018,^8^ and found that during a median follow‐up of 5.4 years (interquartile range [IQR], 0.8–10.8 years), 1730 of 3307 patients (52.3%) had at least 1 cardiac operation or transcatheter intervention in addition to the planned staged surgeries. We found a higher risk of additional cardiac surgery and transcatheter reinterventions between palliative staged surgeries, based on risk factors of the initial stage 1 approach (eg, hybrid versus Norwood); greater patient complexity in terms of noncardiac comorbidities (eg, genetic syndromes); additional cardiac problems (eg, impaired ventricular function); and in children who had already had a more complex operative pathway than standard.^9^

In the current study, we aimed to explore the relationship between early reinterventions and survival beyond 1 year of age in children with f‐SV disease, with the following 2 study questions:

1. What types of additional or off‐pathway interventions were performed in patients with f‐SV in the first year of life?
2. What was the relationship between additional or off‐pathway interventions in the first year of life and outcomes of survival beyond 1 year of age and of Fontan completion in patients with f‐SV disease?

## METHODOLOGY

### Study Design

The current study was a retrospective cohort investigation based on the National Congenital Heart Diseases Audit (NCHDA), with survival status from the Office of National Statistics (ONS).

### Data Sources

We used all records of cardiac surgical procedures and interventional catheters performed in England and Wales between April 1, 2000, and March 31, 2018. During this period, data submission to the NCHDA was mandatory and subject to external data validation. Each procedure record contains several diagnostic and procedure codes from the European Pediatric Cardiac Code (itself a derived Short List of the International Pediatric and Congenital Cardiac Code) (www.ipccc.net).^10^

Patient vital status (dead or alive) was provided at the point of hospital discharge by NCHDA, which obtained this information from treating centers. The age at death for any patient who had died (whether in or out of hospital) was taken from death certification data provided by the ONS, linked to the patient's National Health Service (NHS) number (unique identifier assigned at birth or first contact with the UK health system). For surviving patients, we received from ONS their age when this status was confirmed (November 2020). Any patients who were discharged alive and who had missing life status with ONS were deemed lost to follow‐up and were censored at their most recent discharge age provided by NCHDA.

As the NCHDA is a procedure‐based data set, patients who did not undergo any surgical or interventional cardiac procedures do not appear in the data set.

### Data Approvals

The study was approved by the NCHDA Research Committee and the NHS Healthcare Quality Improvement Partnership (application number 18‐CON‐04), the Stanmore NHS Research Ethics Committee (REC number 18/LO/1688), and the Health Research Authority Confidentiality Advisory Group (CAG number 17/CAG/0071) permitting the use of registry data for specific research purposes without consent. The research data set is available to other researchers under these conditions.

### Inclusions and Exclusions

The inclusion and exclusion criteria are depicted in Figure S1.

#### Inclusions

We included patients with f‐SV congenital heart disease (CHD) who were born between April 2000 and March 2018.

#### Exclusions

We excluded patients born before April 2000 to ensure that the complete procedure history was present. We excluded non‐NHS patients and patients from overseas, Scotland, and Northern Ireland, because life status data are collected by ONS for patients from England and Wales only. Based on agreement from 2 clinicians (K.B. and R.F.), we excluded 197 patients: 97 who on closer inspection were found to have biventricular heart disease and 100 who had an infeasible procedure sequence or clinically significant missing data, which meant that a reliable patient history could not be ascertained.

### Cohort Definition

We categorized patients with f‐SV based on whether they survived free of transplant beyond 1 year of age. We described the group of children with f‐SV who died, had a heart transplant, or were censored before age 1 year. We created a cohort for analysis of the outcomes involving children who had recorded transplant‐free survival after the age of 1 year, to allow exposure to additional interventions in infancy (which was the exploratory variable).

### Outcomes

Outcomes were only evaluated in the children with transplant‐free survival to age 1 year (and older). The primary outcome was transplant‐free survival, and the secondary outcome was Fontan completion. Heart transplant was considered a failure.

### CHD Types

We identified the following CHD types as detailed in Table S1: classic hypoplastic left heart syndrome (HLHS);^11^, ^12^ F‐SV with a double‐inlet left ventricle, non‐HLHS mitral atresia, and tricuspid atresia; unbalanced common atrioventricular septal defect; only one fully well‐developed ventricle and atrial isomerism^13^, ^14^; and major primary congenital heart diagnoses with f‐SV circulation where, due to the presence of a hypoplastic ventricle or a straddling atrioventricular valve, the management pathway entailed staged procedures along a f‐SV pathway.^15^ Patients were assigned to f‐SV subtypes for which there were at least 100 patients, combining rarer conditions into “other f‐SV group,” using a hierarchical approach: HLHS, f‐SV with atrial isomerism, double‐inlet left ventricle, tricuspid atresia, mitral atresia without HLHS, unbalanced atrioventricular septal defect, pulmonary atresia without other complex features but with f‐SV, and “other f‐SV.”

### Procedure Types: Planned Treatment Pathway

We considered only cardiac surgery and transcatheter interventions in our analyses. We first identified procedures before or on the planned treatment pathway, as detailed in Table S2.

*Prepathway procedures*: interventions including both surgery and catheter intervention types that occurred after the child's birth (fetal procedures were not in the data set) and before the first staged surgery.^12^, ^14^

*Procedures on the planned treatment pathway* ^3^, ^15^: palliative first‐stage procedures (stage 1) including surgical, hybrid, and catheter types; second‐stage procedures of bidirectional superior cavopulmonary (Glenn) anastomosis or a combination of aorto‐pulmonary amalgamation and augmentation with construction of a bidirectional superior cavopulmonary (Glenn) anastomosis (comprehensive stage 2) surgery; and third stage of total cavopulmonary connection procedures (Fontan). We note that in certain patients, the occurrence of >1 “palliative first‐stage procedure” may occur, but, for the purpose of our study analysis, we allowed only a single stage 1 to be counted on the “treatment pathway.”

*Concurrent procedures*: by clinical consultation, we identified specific concurrent procedures that may occur alongside each staged procedure and indicate that a more complex operation was undertaken, eg, pulmonary venous anomaly repair, atrioventricular valve repair, pulmonary repair, and pacemaker placement at the time of stage 1, 2, and 3; either or both Damus redo or arch repair at the same time as stage 2 and 3; and maze operation at the same time of stage 3 (Table S3).

### Procedure Types: Additional Procedures or Reinterventions (the Exposure of Interest)

“Additional procedures/reinterventions” were all cardiac procedures for residual or recurrent lesions, over and above the treatment pathway. We categorized the additional procedures into surgery (inclusive of operations and procedures involving both surgery and catheters, ie, hybrid types) and catheter interventions (inclusive of interventional catheterizations and procedures involving electrophysiology interventions via catheterization) as detailed in Table S4.^16^ Within our analyses, children were permitted to have only one stage 1, 2, or 3 procedure as part of the planned treatment pathway.

### Risk Factors

We used the counts of additional surgeries and additional catheter interventions during the first year of patients' lives (ie, none, 1, or ≥2) as the main exploratory risk factors. Our data set also included the following patient‐level variables, which we used to adjust for case mix: as previously defined,^17^ f‐SV diagnostic subtype, congenital extracardiac comorbidities (eg genetic syndrome, congenital lung anomalies), sex, prematurity (birth at gestation <37 weeks), and additional cardiac risk factors (eg, impaired ventricular function and pulmonary hypertension^18^). We derived procedure‐based risk factors at the first cardiac procedure: age at procedure, acquired comorbidities (eg, necrotizing enterocolitis and renal failure^18^), increased severity of illness (eg, need for preoperative ventilation or presence of preoperative shock^18^), and low weight (<2.5 kg). We calculated weight‐for‐age *z* scores based on British Growth Reference^19^ and considered those outside the range of +5 and −8 as clinically anomalous and their weight treated as missing. Moreover, we used the occurrence of prepathway procedures (any type), the subtype of stage 2, and the concurrent surgery at stage 1 and 2 as risk factors.

### Statistical Analysis

Based on annual NCHDA reports of external validation, data on diagnosis, procedures, and survival status are of excellent quality from the year 2000; however, data quality for certain clinical variables (severity of illness, acquired comorbidities, and congenital comorbidities) was poor initially and improved after 2009 (when the processes for data quality were changed^20^). Therefore, we included as the time era factor a division of pre‐/post‐2009 in the models.

The ONS data containing patient survival status were received 30 months after the most recent procedure reported in the NCHDA data (due to the time required for regulatory processes), so procedures undertaken during this time gap may have been missed. We therefore used patients’ status as of March 31, 2018, to group patients based on their transplant‐free survival of either longer or <1 year and calculate the status of Fontan surgery completion. Moreover, we reviewed patients who were recorded as alive in ONS but who had not undergone the next expected pathway in surgical management. Based on clinical consensus (K.B. and R.F.), we censored 68 patients at their last postprocedure discharge age in the registry.

Descriptive statistics are presented as number and percentage or as median and interquartile range (IQR). We compared patient and procedure‐based variables in the cohort applicable to children who experienced <1 year of recorded transplant‐free survival since birth with the values for children with >1 year of transplant‐free survival since birth using Fisher exact test or 2‐sample *t* test as appropriate.

Survival analysis was performed using the Kaplan–Meier approach for children who had at least 1 year of transplant‐free follow‐up since birth, with death or heart transplant after 1 year of age representing failure. We developed univariable and multivariable Cox proportional hazards models to investigate the association between the patient's transplant‐free survival time after the age of 1 year and the count of additional surgeries and catheter interventions during the first year of life, adjusting for the other prespecified risk factors. Unadjusted hazard ratio (HR) and adjusted HR (aHR) estimates with 95% CIs are presented for each of the risk factors.

For the outcome of Fontan completion in children who had transplant‐free survival beyond 1 year of age, competing risk analysis was performed because the competing events, ie, death or heart transplant before Fontan, preclude the occurrence of Fontan surgery. Cumulative incidence function^21^ was used to describe the incidence of Fontan completion by additional interventions that had occurred in the first year of life. Univariable and multivariable Fine and Gray subdistribution hazard models^21^, ^22^ were performed to investigate the association between the cumulative incidence function of Fontan completion and the occurrence of additional interventions in the first year of life, adjusting for the prespecified covariates. Results are presented as subdistribution HR and adjusted subdistribution HR (aSHR) with 95% CI.

In both the Cox proportional hazard and Fine and Gray subdistribution hazard models, robust standard errors were computed to allow for clustering within the center. We used multiple imputation by logistic regression including all risk factors to assign the missing binary values for low weight (2.1%) and estimates were combined by using Rubin rules.^23^ In addition, time‐varying factors were fitted for risk factors where the proportional hazards assumption was not valid (ie, test of proportional hazard assumption using Schoenfeld residuals *P*<0.05). The association between additional interventions and other risk factors was explored using Fisher exact test and, for those where the association was significant (*P*<0.05), the interaction between the risk factor and additional interventions was examined by including the interaction terms in the model. Sensitivity analysis was performed by removing the 2 risk factors (increased severity of illness and acquired comorbidity at the time of the first operation) that were most poorly populated for data quality in the early era for NCHDA before 2009 from both models.^2^

All statistical analyses were performed with Stata 15 software (StataCorp LLC).

## RESULTS

### Study Population

**Figure 1:**
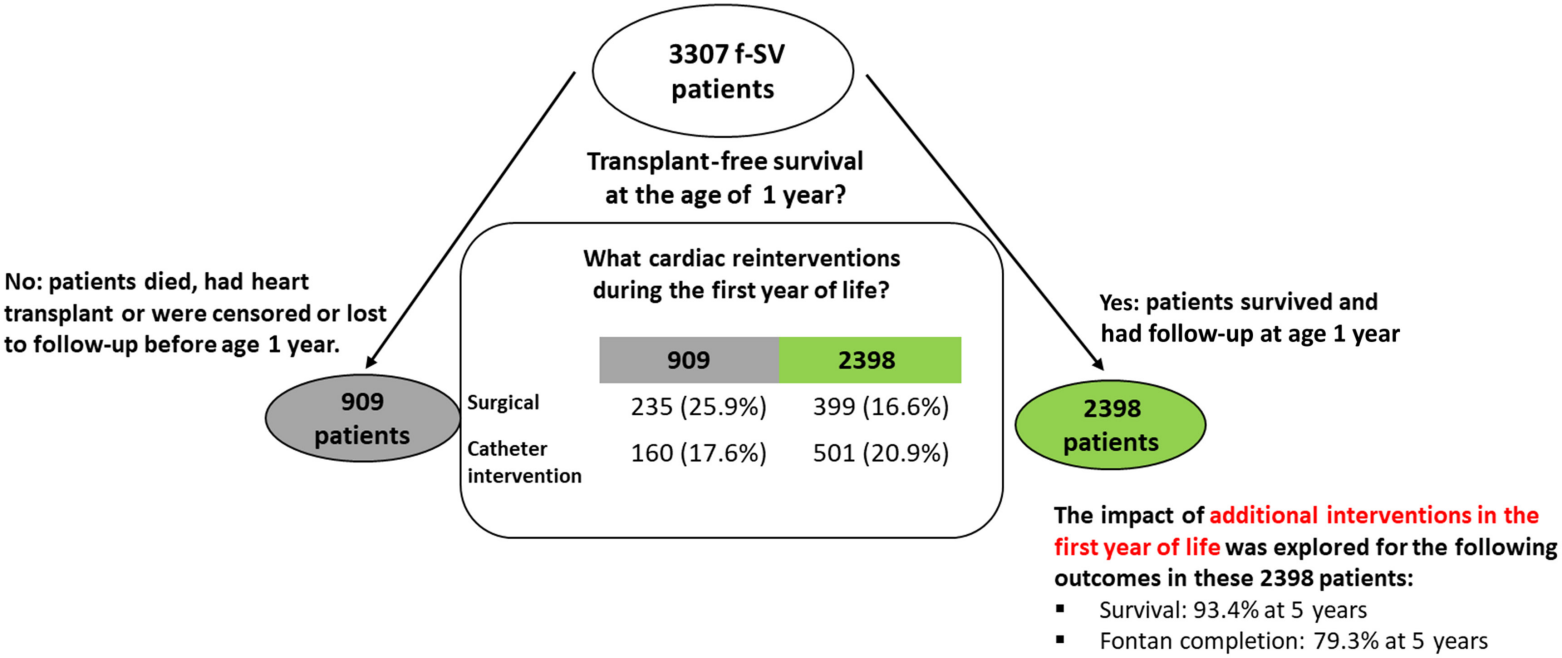
Flow chart of the study cohort and outcomes for patients with functionally single ventricle (f‐SV).

From the population of 56 039 patients in the NCHDA, 3307 (5.9%) patients with f‐SV were identified, as depicted in Figure S1. Among 3307 patients with f‐SV, 909 (27.5%) had <1 year of transplant‐free follow‐up since birth and 2398 (72.5%) had a transplant‐free follow‐up beyond 1 year of age (Figure 1).

Of those 909 children with <1 year of recorded transplant‐free survival by March 31, 2018, 755 had died before the age of 1 year, at a median age of 50 days (IQR, 15–126 days), including 2 who died after a heart transplant. In addition, 11 had undergone heart transplant (survived) and 143 were censored before reaching 1 year of age, of whom 84 were <1 year old when data were harvested and 59 were censored at last discharge age.

The 909 children with transplant‐free follow‐up <1 year since birth, were more complex than the 2398 with follow‐up beyond infancy. As shown in Table 1, the proportions of patients with higher‐risk f‐SV subtypes of HLHS and unbalanced atrioventricular septal defect^2^ were 71.4% (n=649) and 35.4% (n=848), respectively (*P*<0.001), and they were more likely to have low weight (142 [15.6%] versus 197 [8.2%] *P*<0.001), acquired comorbidity (68 7.5%) versus (91 [3.8%] *P*<0.001), and critical illness (155 [17.1%] versus 229 [9.5%] *P*<0.001) at their first intervention.

**Table 1:** Characteristics of Patients With f‐SV Disease

Patient factor	Patients with transplant‐free survival <1 y since birth (n=909)	Patients with transplant‐free survival beyond 1 y of age (n=2398)	*P* value*
Noncardiac variables			
Sex: male	526 (57.9)	1411 (58.8)	0.64
Prematurity	59 (6.5)	140 (5.8)	0.51
Congenital noncardiac comorbidity	156 (17.2)	397 (16.6)	0.68
Low‐weight baby (<2.5 kg) at first procedure	142 (15.6)	197 (8.2)	<0.001
Acquired comorbidity at first procedure	68 (7.5)	91 (3.8)	<0.001
Increased severity of illness at first procedure	155 (17.1)	229 (9.5)	<0.001
Age at first procedure, d	6 (4–11)	8 (5–42)	<0.001
Recent data: born after April 2009	494 (54.3)	1203 (50.2)	0.03
f‐SV subtype			
HLHS	565 (62.2)	701 (29.2)	<0.001
f‐SV with atrial isomerism	48 (5.3)	195 (8.1)	<0.001
Double‐inlet left ventricle	39 (4.3)	289 (12.1)	<0.001
Tricuspid atresia	86 (9.5)	362 (15.1)	<0.001
Mitral atresia without HLHS	22 (2.4)	90 (3.8)	0.07
Unbalanced atrioventricular septal defect	84 (9.2)	147 (6.1)	<0.001
Pulmonary atresia without other complex features but with f‐SV	8 (0.9)	130 (5.4)	<0.001
Other f‐SV types	57 (6.3)	484 (20.2)	<0.001
Additional cardiac risk factor (any time point)	69 (7.6)	165 (6.9)	0.49
Prestage 1 procedure (any type)	93 (10.2)	246 (10.3)	0.99
Stage 1 and subtypes (at first stage 1)			
No stage 1 procedure	51 (5.6)	340 (14.2)	<0.001
Norwood type	513 (56.4)	855 (35.7)	<0.001
Isolated arch repairs	63 (6.9)	125 (5.2)	0.06
Hybrid procedure with bilateral banding and ductal stent	72 (7.9)	65 (2.7)	<0.001
Secure pulmonary blood flow (arterial shunt or right ventricular outflow tract procedure or ductal stent)	132 (14.5)	697 (29.1)	<0.001
Pulmonary arterial band	78 (8.6)	316 (13.2)	<0.001
Stage 1 with concurrent surgery	57 (6.3)	100 (4.2)	0.01
Stage 2 and subtypes			
No stage 2 procedure	751 (82.6)	121 (5.0)	N/A
Glenn	143 (15.7)	2127 (88.7)	N/A
Comprehensive stage 2	15 (1.7)	150 (6.3)	N/A
Stage 2 with concurrent surgery	76 (8.4)	875 (36.5)	N/A

Low weight includes imputed data, 12 (1.3%) and 56 (2.3%) for patients with and follow‐up duration, and longer than 1 year since birth, respectively. Data are expressed as number (percentage) or median value (interquartile range).

* Test of association between patients' factors and follow‐up duration (or beyond 1 year of age): Fisher exact test and 2‐sample *t* test were performed when appropriate. f‐SV indicates functionally single‐ventricle; HLHS, hypoplastic left heart syndrome; and N/A, not available.

Question 1: What types of additional or off‐pathway interventions were performed in patients with f‐SV in the first year of life?

#### Procedure Pathways in f‐SV

**Figure 2:**
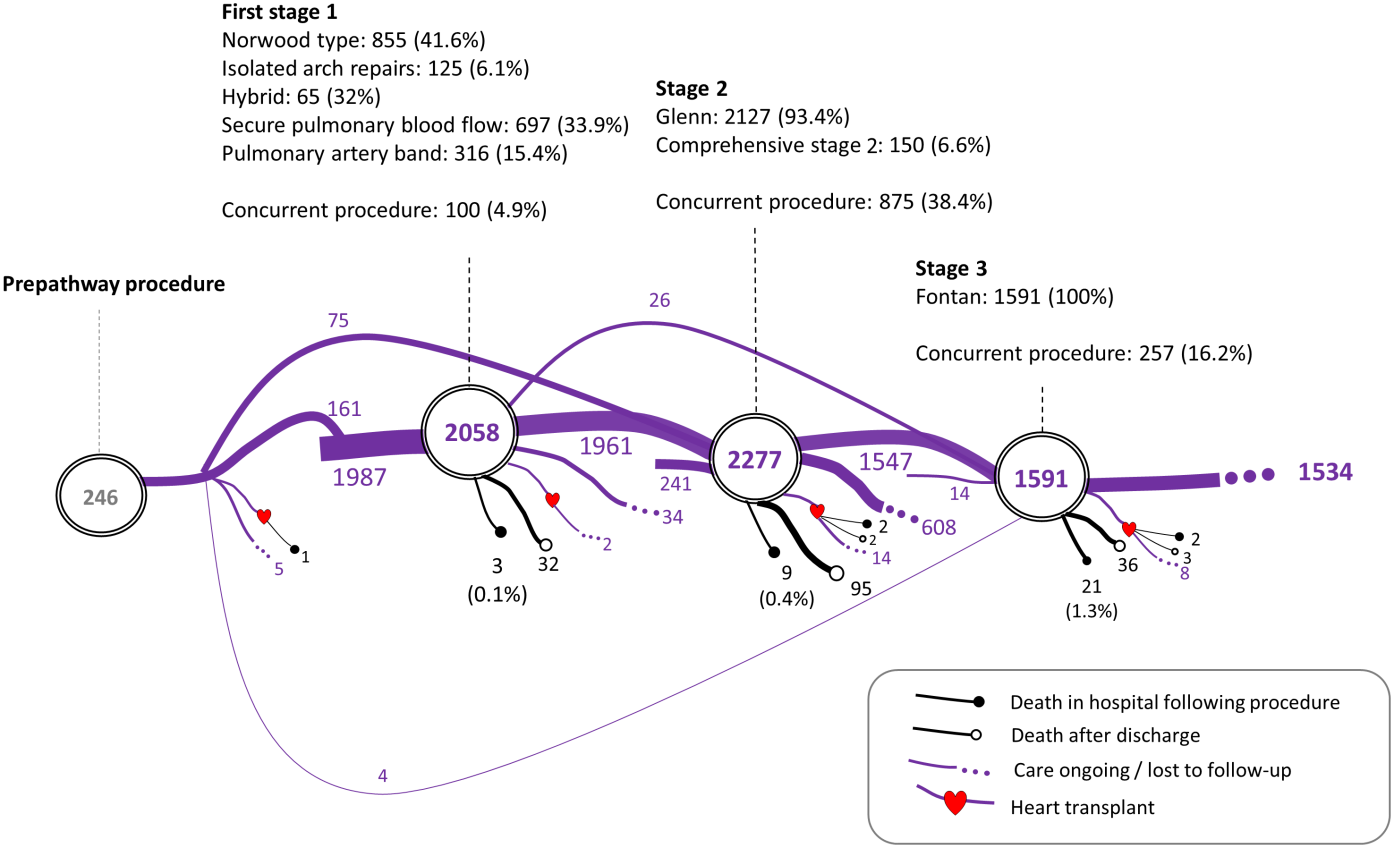
Planned treatment pathways and survival outcomes for 2398 patients with functionally single‐ventricle (f‐SV) disease with transplant‐free survival beyond 1 year of age. The subtypes of staged procedure are presented as number (percentage), where the ratio is computed based on the number of patients who had stage 1, 2, or 3 procedure. Patients with transplant‐free survival <1 year since birth (died, had heart transplant, or were censored before the age of 1 year) were not included.

To answer question 1, we first depicted the procedures that occurred before and during planned treatment pathways. We display pathway procedures for the 2398 patients with transplant‐free follow‐up beyond 1 year of age in Figure 2, which captures the number of patients who had undergone each surgical stage at the time the data set was extracted but does not display the additional procedures (study outcome). The pathway diagram for the 909 patients with transplant‐free survival of <1 year since birth is presented separately in Figure S2 and we compare the prepathway and stage 1 procedure types among children in the 2 groups in Table 1.

The proportion of patients with a prepathway procedure, such as balloon atrial septostomy, was similar in both groups of children (93 [10.2%] and 246 [10.3%]). As expected, given the greater prevalence of HLHS among children who had transplant‐free survival of <1 year since birth, they had more Norwood (513 [56.4%] versus 855 [35.7%] *P*<0.001) and hybrid (72 [7.9%] versus 65 [2.7%] *P*<0.001), stage 1 subtypes than the children who had follow‐up beyond 1 year of age. Conversely, stage 1 procedures to secure pulmonary blood flow were less common among children with transplant‐free survival of <1 year since birth (132 [14.5%] versus 697 [29.1%] *P*<0.001), as were isolated pulmonary arterial bands (78 [8.6%] versus 316 [13.2%] *P*<0.001). Also reflecting their complexity, children with transplant‐free survival of <1 year since birth were more likely to have had a stage 1 (858 [94.4%] versus 2058 [85.8%] *P*<0.001) and concurrent surgery such as pulmonary arterial repair with their stage 1 (57 [6.3%] versus 100 [4.2%] *P*=0.01) compared with the children with follow‐up beyond 1 year of age.

#### Additional or Off‐Pathway Procedures in the First Year of Life

The most common reinterventions during the first year of life are presented in Table 2 (for details by f‐SV subtype see Table S5). The most common surgery was modified right Blalock interposition shunt in 37 of 909 (4.1%) children with <1 year of follow‐up since birth and 80 of 2298 (3.3%) of children with follow‐up beyond 1 year of age, and the most common catheter reintervention was balloon dilation of aortic recoarctation in 32 of 909 (3.5%) children with <1 year of follow‐up since birth and 143 of 2298 (6.0%) children with follow‐up beyond 1 year of age.

**Table 2:** Frequency of Additional Procedures or Off‐Pathway in the First Year of life for All Patients With f‐SV Disease

Top 5 most common additional surgeries and catheter interventions in 2398 patients with transplant‐free survival beyond 1 y of age		
Additional surgery	Modified right Blalock interposition shunt	80 (3.3)
	Pulmonary arterioplasty/reconstruction	68 (2.8)
	Atrial septectomy	61 (2.5)
	Pulmonary trunk band (PA band)	53 (2.2)
	Procedure involving constructed cardiac conduit‐shunt	27 (1.1)
Catheter intervention	Balloon dilation of aortic recoarctation	143 (6.0)
	Balloon atrial septostomy by pull back (Rashkind)	90 (3.8)
	Stent placement in cardiac conduit	49 (2.0)
	Balloon dilation of left pulmonary artery	46 (1.9)
	Stent placement in left pulmonary artery	37 (1.6)

Top 5 most common additional surgeries and catheter interventions in 909 patients with transplant‐free survival <1 y since birth		
Surgery	Modified right Blalock interposition shunt	37 (4.1)
	Norwood type procedure	33 (3.7)
	Procedure involving constructed cardiac conduit‐shunt	30 (3.3)
	Pulmonary trunk band (PA band)	24 (2.7)
	Atrial septectomy	21 (2.3)
Catheter intervention	Balloon dilation of aortic recoarctation	32 (3.5)
	Balloon atrial septostomy by pull back (Rashkind)	28 (3.1)
	Stent placement in cardiac conduit	22 (2.4)
	Balloon dilation of left pulmonary artery	18 (2.0)
	Transluminal occlusion of systemic‐to‐pulmonary collateral arteries (major aortopulmonary collateral arteries) with coil device	14 (1.6)

Data are expressed as number (percentage). Procedures may arise more than once in the same patient. f‐SV indicates functionally single‐ventricle; and PA, pulmonary artery.

The detailed numbers of additional surgeries and interventional catheters are provided in Table S6. Additional procedures in infancy occurred in 323 of 909 (35.5%) children with transplant‐free survival of <1 year since birth and 756 of 2398 (31.5%) children with transplant‐free survival beyond 1 year of age (*P*=0.031).

Children with transplant‐free survival of <1 year since birth were more likely to have had an additional surgery (235 of 909 [25.9%] versus 399 of 2398 [16.6%], *P*<0.001), whereas children with transplant‐free survival beyond 1 year of age were slightly more likely to have had an additional catheter intervention (501 of 2398 [20.9%] versus 160 of 909 [17.6%], *P*=0.037). Multiple reinterventions in the first year of life were more likely in children with transplant‐free survival of <1 year since birth (122 of 909 [13.4%] versus 252 of 2398 [10.5%], *P*=0.019) and this difference was accounted for by more multiple surgeries in the children with transplant‐free survival of <1 year since birth.

Question 2: What was the relationship between additional interventions in the first year of life and the outcomes of survival beyond infancy and of Fontan completion?

#### Transplant‐Free Survival in Children Reaching 1 Year of Age

**Figure 3:**
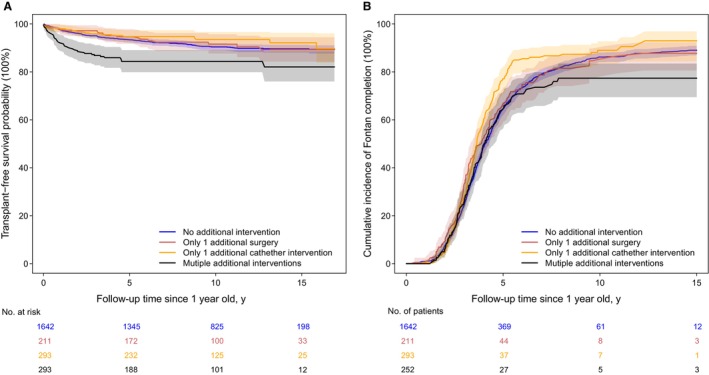
Kaplan–Meier curves and cumulative incidence of Fontan completion showing survival and Fontan completion in children with transplant‐free survival beyond 1 year of age. (**A**) Kaplan–Meier curves for transplant‐free survival rate and (**B**) cumulative incidence of Fontan completion with 95% CI in patients who had no additional intervention during the first year of life, had 1 additional surgery only, had 1 additional catheter only, and had multiple additional interventions, for 2398 patients with transplant‐free survival beyond 1 year of age. Patients with transplant‐free survival <1 year since birth (died, had heart transplant, or were censored before the age of 1 year) were not included.

Over a median follow‐up of 10.7 years (IQR, 6.7–14.9 years; maximum, 20.6 years) the 5‐year transplant‐free survival rate was 93.4% (95% CI, 92.4%–94.4%) among children who survived infancy transplant‐free. Heart transplant was rare after the age of 1 year, occurring in only 34 (1.4%). We present the Kaplan–Meier curves by number of additional interventions in infancy for children with follow‐up beyond 1 year of age in Figure 3A. The survival curves were similar among patients with no additional interventions, 1 additional surgery only, and 1 additional catheter intervention only, while patients with multiple reinterventions (any type) had a consistently lower survival probability over time.

#### Risk Factors for Mortality in Children Reaching 1 Year of Age

In the univariable analysis, we found that the occurrence of more than 1 surgery or catheter intervention in infancy was associated with greater mortality, ie, HR, 3.55 (95% CI, 1.81–6.95; *P*<0.001) and 2.35 (95% CI, 1.60–3.47; *P*<0.001), respectively (Table S7). In the multivariable analysis, after considering all other predefined risk factors, we found that the occurrence of multiple surgeries in infancy remained significant (aHR, 3.93 [95% CI, 1.87–8.27]; *P*<0.001), but multiple catheter interventions were no longer significantly linked to mortality (aHR, 1.49 [95% CI, 0.92–2.42]; *P*=0.11) (Table 3). Patients who had only 1 additional surgery or 1 additional catheter intervention in infancy had no extra risk of mortality in either model (Table 3; Table S6).

**Table 3:** Survival and Fontan Completion Among Children With Transplant‐Free Survival Beyond 1 Year of Age

Risk factors	Survival outcome beyond age 1 y	Fontan completion
	aHR (95% CI)	aSHR (95% CI)
No. of additional surgeries in the first year of life (reference: no additional surgery)		
1	1.06 (0.74–1.53)	0.99 (0.78–1.26)
≥2	3.93 (1.87–8.27)^‡^	0.81 (0.48–1.35)
No. of additional interventional catheters in the first year of life (reference: no additional catheter intervention)		
1	0.76 (0.39–1.50)	1.01 (0.88–1.17)
≥2	1.49 (0.92–2.42)	0.71 (0.52–0.96)*
f‐SV subtypes (reference: HLHS)		
f‐SV with atrial isomerism	1.62 (1.00–2.61)*	0.19 (0.09–0.43)^‡^
f‐SV with atrial isomerism×follow‐up time	…	1.26 (1.13–1.41)^‡^
Double‐inlet left ventricle	0.42 (0.16–1.09)	0.86 (0.62–1.18)
Tricuspid atresia	0.41 (0.23–0.76)^†^	1.31 (1.22–1.39)^‡^
Tricuspid atresia×follow‐up time	…	0.34 (0.14–0.78)*
Mitral atresia without HLHS	0.54 (0.23–1.27)	1.26 (1.07–1.47)^†^
Mitral atresia without HLHS×follow‐up time	…	0.65 (0.48–0.88)^†^
Unbalanced atrioventricular septal defect	1.61 (0.90–2.87)	0.67 (0.55–0.81)^‡^
Pulmonary atresia without other complex features but with f‐SV	0.50 (0.22–1.15)	1.18 (1.10–1.28)^‡^
Other f‐SV types	0.76 (0.41–1.40)	0.19 (0.09–0.43)^‡^
Other f‐SV types×follow‐up time	…	1.26 (1.13–1.41)^‡^
Additional cardiac risk factor (reference: none)	0.84 (0.52–1.35)	0.67 (0.56–0.81)^‡^
Additional cardiac risk factor×follow‐up time	1.16 (1.08–1.25)^‡^	…
Surgical variables		
Prestage 1 procedure (any type) (reference: none)	0.86 (0.54–1.38)	0.95 (0.79–1.15)
Stage 1 with concurrent surgery (reference: none)	1.17 (0.67–2.04)	0.88 (0.64–1.21)
No stage 2 procedure (reference: Glenn)	25.80 (13.28–50.10)^‡^	0.49 (0.31–0.78)^†^
No stage 2 procedure×follow‐up time	0.60 (0.48–0.74)^‡^	…
Comprehensive stage 2 (reference: Glenn)	0.99 (0.60–1.62)	1.22 (1.00–1.49)
Stage 2 with concurrent surgery (reference: none)	1.07 (0.78–1.47)	0.91 (0.80–1.04)
Noncardiac variables		
Male (reference: female)	0.92 (0.69–1.24)	1.12 (0.98–1.28)
Prematurity (reference: born ≥37 wk of gestation)	0.60 (0.25–1.45)	1.03 (0.84–1.25)
Congenital noncardiac comorbidity (reference: none)	0.64 (0.38–1.08)	0.45 (0.35–0.59)^‡^
Congenital noncardiac comorbidity×follow‐up time	1.14 (1.03–1.26)^†^	1.14 (1.07–1.21)^‡^
Low weight at first procedure (reference: ≥2.5 kg)	1.63 (1.02–2.60)*	0.94 (0.80–1.10)
Acquired comorbidity at first procedure (reference: none)	1.21 (0.83–1.78)	0.91 (0.64–1.29)
Increased severity of illness at first procedure (reference: none)	1.19 (0.68–2.07)	0.83 (0.64–1.07)
Age at first procedure, y	0.88 (0.73–1.05)	0.63 (0.55–0.73)^‡^
Age at first procedure×follow‐up time, y	…	1.04 (1.03–1.06)^‡^
Born after April 2009 (reference: born before April 2009)	0.84 (0.51–1.38)	1.18 (0.99–1.41)

Patients with transplant‐free survival <1 year since birth (died, had heart transplant, or were censored before the age of 1 year) were not included in the model. Center‐clustered standard errors computed. *P* values: *0.05, ^†^0.01, and ^‡^0.001. Interpretation of coefficients for covariates with time‐varying interaction term. Consider A as a categorical covariate and the hazard regression coefficients for A and the time interaction term A×follow‐up time (years from birth) are expressed as β and γ, respectively. The estimation of “β+γ*follow‐up time” represents the change in the expected log of the hazard ratio relative to the reference. In the table, we report the baseline hazard ratio and time‐changing hazard ratio by exp (β) and exp (γ). A value of exp (γ) >1 indicates the mortality risk will increase with time, compared with the reference group, and vice versa. aHR indicates adjusted hazard ratio; aSHR, adjusted subdistribution hazard ratio; HLHS, hypoplastic left heart syndrome; and f‐SV, functionally single‐ventricle.

Considering case mix factors in the multivariable model, compared with HLHLS, f‐SV with atrial isomerism was at higher risk of mortality (aHR, 1.62 [95% CI, 1.00–2.61]; *P*=0.04) and tricuspid atresia was at lower risk of mortality (aHR, 0.41 [95% CI, 0.23–0.76]; *P*=0.004) (Table 3). Children who had no stage 2 (n=121) had a very high risk of mortality (aHR, 25.80 [95% CI, 13.28–50.10]; *P*<0.001), but this risk decreased over time (aHR, 0.60 [95% CI, 0.48–0.74]; *P*<0.001). Children who had additional cardiac risk factors (time interaction aHR, 1.16 [95% CI, 1.08–1.25]; *P*<0.001) and congenital comorbidity (time interaction aHR, 1.14 [95% CI, 1.03–1.26]; *P*=0.009) had an increasing risk of mortality over time. Children with low weight at their first intervention had a higher risk of mortality (aHR, 1.63 [95% CI, 1.02–2.60]; *P*=0.04).

#### Fontan Completion in Patients With Transplant‐Free Follow‐Up Beyond 1 Year of Age

Among the 2398 patients with transplant‐free follow‐up beyond infancy, 1591 (66.3%) had a Fontan operation at a median age of 4.5 years (IQR, 3.7–5.6 years); 147 (6.1%) died or had a heart transplant before Fontan and 660 (27.5%) were censored without undergoing Fontan with a median age of 2.6 years (IQR, 1.2–4.5 years; maximum, 16.9 years). The cumulative incidence of Fontan surgery with 95% CI by number of additional interventions in the first year of life is presented in Figure 3B, and the cumulative incidence of Fontan at 5 years old was 79.3% (95% CI, 77.4%–81.2%).

#### Risk Factors for Fontan Completion

In univariable competing risk models, there was no relationship between the occurrence of additional surgery or catheter intervention in infancy and Fontan completion (Table S7). In multivariable models, after adjustment for other risk factors, patients who had more than 1 additional catheter intervention had a slightly lower likelihood of achieving Fontan (aSHR 0.71 [95% CI, 0.52–0.96]; *P*=0.03) (Table 3). The occurrence of single or multiple additional surgeries in infancy was unrelated to the chance of Fontan surgery completion.

Considering case mix factors in the multivariable model, compared with HLHS, all other f‐SV subtypes except for double‐inlet left ventricle were less likely to have Fontan surgery at an early age and were more likely to achieve Fontan at an older age (follow‐up time interaction terms all >1, except for unbalanced atrioventricular septal defect [see details in Table 3]). In addition, children who had no stage 2 (aSHR, 0.49 [95% CI, 0.31–0.78]; *P*=0.003), additional cardiac risk factors (aSHR, 0.67 [95% CI, 0.56–0.81]; *P*<0.001), and congenital comorbidities (aSHR, 0.45 [95% CI, 0.35–0.59] *P*<0.001; with aSHR congenital comorbidities time interaction factor, 1.14 [95% CI, 1.07–1.21] *P*<0.001) all had a lower likelihood of achieving Fontan. Patients who were older at first intervention were more likely to have their Fontan at an older age (indicated by aSHR in the first year, 0.63 [95% CI, 0.55–0.73]; *P*<0.001; with aSHR time interaction factor, 1.04 [95% CI, 1.03–1.06]; *P*<0.001). Finally, children born after 2009 had a borderline higher chance of achieving Fontan (aSHR, 1.18 [95% CI, 0.99–1.41]; *P*=0.06).

In both the Cox proportional hazard and Fine and Gray subdistribution hazard models, we conducted subgroup analysis via fitting separate models of including the interaction terms between additional procedures and confounders (risk factors that were significantly associated with additional procedures, Fisher exact test *P*<0.05) (Table S8). There was no evidence that the relationship between additional interventions with survival and Fontan completion was associated with the measured confounders, ie, no lower values in Akaike information criterion or the Bayes information criterion were observed when incorporating these interactions in the model. In addition, in the sensitivity tests that excluded the 2 covariates with the least reliability in early data (severity of illness and acquired comorbidity) from both models, the overall results remained unchanged.

## DISCUSSION

### Summary of Findings

We report a novel population‐based analysis of children who started therapy for f‐SV disease, in which we explored the relationship between cardiac reinterventions in the first year of life and children's subsequent outcomes. Unsurprisingly, children who had no follow‐up beyond infancy, most of whom died, were more complex, dominated by HLHS and other high‐risk features.^2^ The most common types of reinterventions that occurred in both groups of children were redo modified Blalock‐Taussig‐Thomas shunts, balloon dilatation of recoarctation, procedures to open the atrial septum, and pulmonary artery interventions. However, children who had no follow‐up beyond infancy were more likely to have additional surgical arch reconstruction procedures, eg, Norwood or Damus, reflecting the predominance of HLHS. Children with no follow‐up beyond infancy were overall more likely to have needed additional intervention in the first year of life, and this was accounted for by a greater proportion of additional surgery, both single and multiple. When we considered the exposure of additional interventions in the first year of life on outcomes beyond infancy, we found that multiple surgeries were strongly linked to the outcome of mortality (albeit not to the outcome of Fontan completion), and that multiple catheter interventions were linked to lower chances of Fontan completion being achieved. Only 3.5% of children with follow‐up beyond infancy had multiple surgeries in the first year of life, and these children experienced a high risk of death after the age of 1 year; these findings may have obscured any relationship between this exposure and the outcome of Fontan completion.

### Context and Interpretation

We have previously reported from our population‐based f‐SV cohort that long‐term survival is strongly linked to case complexity^2^ and that reinterventions are linked to specific factors of the hybrid pathway (which in the United Kingdom has been used for the highest‐risk babies^12^), increased severity of illness at stage 1, and when there are additional cardiac risk factors (impaired ventricular function or raised pulmonary vascular resistance documented at the time of any procedure).^9^ Then, once one additional procedure is needed, this carries a knock‐on risk of subsequent additional procedures at later stages.^9^ This might explain how a difficult start for a baby, due to initial f‐SV morphology and severity of illness, can contribute to multiple surgeries being required early in life, and the combination of case mix features and multiple reinterventions is likely to contribute in combination to subsequent mortality.

This combined effect of both severe case mix and high early reintervention burden might be pertinent for babies who had a hybrid pathway, in whom mortality was particularly high in this study. We note that a recent systematic review based on 699 patients that compared hybrid and Norwood stage 1 types in patients with HLHS found 1.48 times higher risk of reinterventions with the hybrid procedure,^24^ and follow‐up of these children could shed further light on outcomes.

The children with additional cardiac risk factors were overrepresented in the group who had >1 year of follow‐up, and, in the cohort surviving infancy, those with additional cardiac risk factors had higher mortality and a lower likelihood of Fontan completion. This is an example of how “not all reinterventions are equivalent,” given that Glenn and Fontan takedowns and pacemakers were the most common reinterventions after stages 2 and 3 among children with additional cardiac risk factors, emphasizing their complexity and poorer prognosis, as has been shown in other studies.^25^

Among children with transplant‐free survival longer than 1 year, 121 had no stage 2 procedure, and these children had a very high risk of mortality in the first year of follow‐up (between the ages of 1 and 2 years), later with only 44 subsequently reaching Fontan. These 121 patients were more likely to have multiple surgeries (6.6%) and multiple catheters (6.6%) during infancy, although not statistically significant due to a relatively small sample size. Based on the data sources available, we do not know the reasons for these poor outcomes, but we speculate that the high early mortality may represent children who were not fit to proceed to stage 2 and then died, as well as potentially some unanticipated interstage deaths.

There are very few population‐based data sets with which to compare our data to assess the potential for generalizability. An exception is ANZFR (Australia and New Zealand Fontan Registry), although inclusion in this registry is conditional on survival to Fontan. ANZFR reported pulmonary arterial augmentation in 3.3% of patients following stage 2 and 7.4% at the time of stage 3.^7^ Our study cohort had similar rates of pulmonary arterial interventions: 2.5% after stage 2 and 7.4% at the time of stage 3. The Australia‐New Zealand study found no link between pulmonary arterial reinterventions and survival after Fontan over this timeframe of follow‐up.^7^ The majority of pulmonary arterial interventions in our cohort were transcatheter and we found that a single catheter reintervention was more common among children who had >1 year of follow‐up than in the group who had no follow‐up beyond infancy, and postinfancy outcomes were similar to children who needed no reinterventions. It is possible that these surviving children had more opportunities to undergo cardiac catheterization (than those who died). We found that multiple catheter interventions in infancy was linked to slightly poorer chances of Fontan completion, which might reflect pulmonary arterial problems in a subset of children.

A further albeit imperfect comparator population is children randomized in the Pediatric Heart Network's SVR (Single Ventricle Reconstruction) trial, who had stage 1 surgeries between 2005 and 2008 and achieved 69% freedom from death or transplant at 12 months.^26^ In our population‐based cohort among children with HLHS who underwent the Norwood pathway, there was 62.7% freedom from death or transplant at 12 months. Among our whole f‐SV population, transplantation was uncommon, performed in 47 (1.4%) children. Although the population is not directly comparable, this can be considered against the higher rate of 22 (4%) transplants over a similar age range in the SVR trial groups.^5^ Children who require multiple early reinterventions could represent a subset who might benefit from a review of suitability for transplantation at a young age.

### Strengths and Limitations

This was a registry‐based study reflecting practice in England and Wales and is limited by data quality. The data for CHD and surgical variables in NCHDA are of very high quality during the entire study period; however, the preoperative noncardiac risk variables were of poorer quality before 2009, due to processes within the audit. Sensitivity tests were performed by removing the 2 covariates with the least reliable early data (severity of illness and acquired comorbidity) from the Cox regression and Fine and Gray subdistribution hazard models, and, in doing so, the overall results did not change. As mentioned, although we considered 2009 as the era cut point in our models, this only partially accounts for this limitation.

Our analysis of broader trends in the f‐SV population was limited in this study, and, for context, in Figures S3 through S5 we present 3 charts that depict by birth year, the proportions of children with case mix factors, the stage 1 types, and the survival rates at 1 and 5 years. The main observed changes in the f‐SV population during this period were a rise in antenatal diagnosis and in the proportion of children starting treatment each year who were affected by noncardiac risk factors. We found that patients born after 2009 with a follow‐up beyond 1 year of age exhibited a borderline higher chance of achieving Fontan (aSHR, 1.18 [95% CI, 0.99–1.41]; *P*=0.06), perhaps reflecting recent trends in clinical practice in terms of putting children forward for Fontan. None of our analyses indicated that survival improved over time.

We computed robust standard errors accounting for clustering within center for all models; however, this cannot account for all of the differences between treating centers. The CHD service in England has been highly centralized over the past 20 years, and this contributed to our previous report that the case mix–adjusted 5‐year survival rates for f‐SV were similar between the 10 English centers.^2^ Nonetheless, a full exploration of intercenter differences with respect to reinterventions goes beyond the scope of this study.

At the patient level, we took an inclusive approach, incorporating patients with unusual treatment pathways; hence, some could have been misgrouped due to coding ambiguities. On the other hand, a strength of our study is the novel inclusive approach, which enables exploration of additional procedures among all types of f‐SV disease. Only patients who underwent at least 1 postnatal procedure are captured in NCHDA, but our inclusion of patients who underwent any cardiac intervention provides a more complete picture than more selective studies, eg, those with exclusive focus on specific procedures.

Although rigorously defined, we recognize that counts of additional off‐pathway procedures as a method to identify reinterventions is a simplification. For example, we grouped second “stage 1–type procedures” (which accounted for 67% of surgery and 15.5% of interventional catheters at this stage) as reinterventions, acknowledging that in a subset of cases, these could be viewed as part of planned treatment, such as in 28 children who had hybrid for HLHS pathway then a Norwood operation. Moreover, there are qualitative clinical differences between reintervention types, which cannot be accounted for in our analyses. When considering the degree to which reinterventions may be planned, it is important to acknowledge that institutional protocols may vary with respect to treatment strategies and to prestage evaluation. In addition, centers that routinely undertake cardiac catheterization may be more likely to undertake preemptive catheter intervention before the stage than centers assessing with magnetic resonance that may be more likely to intervene at the time of staged procedure. There were 28 (1.2%) children who had a stage 2 takedown and 5 (0.3%) children who had a Fontan takedown within the whole cohort; these procedures were counted as “reinterventions,” although we recognize this is a limited description. Overall, we did not find evidence that the effect of reinterventions differs among various subgroups within our study cohort, given the potentially limited sample size.

## CONCLUSIONS

For children with f‐SV disease, additional procedures are common, representing an important component of their journey, and are a means by which attempts are made to optimize the f‐SV circulation. Unfortunately, children who need multiple additional interventions in the first year of life, especially surgeries, have poorer outcomes. Children who have the most severe disease at the start of their journey and are burdened by multiple surgeries had the poorest outcomes.

## Sources of Funding

This study was funded by the British Heart Foundation (project grant number PG/17/88/33401). V.T. and K.L.B. received support from the NIHR Biomedical Research Centre at Great Ormond Street Hospital.

## Disclosures

None.

## Supporting information

Tables S1–S8
Figures S1–S5

## Footnotes

1 See Editorial by Puri and Denfield.
