# Supplementary material for: Retrospective Cohort Study of Additional Procedures and Transplant‐Free Survival for Patients With Functionally Single Ventricle Disease Undergoing Staged Palliation in England and Wales
Source: J Am Heart Assoc. 2024 Jul 3;13(14):e033068. doi: 10.1161/JAHA.123.033068 (PMC11292744; doi:10.1161/JAHA.123.033068)

SUPPLEMENTAL MATERIAL

Table S1. Definition of functionally single ventricle (f-SV) congenital heart disease (CHD) patients.

f-SV Disease Types	Definition of CHD Type
**Classic hypoplastic left heart syndrome (HLHS)**	CHD with a small left ventricle, left sided valvar stenosis or atresia, normally related great arteries and no common atrioventricular junction based on diagnostic and procedure codes as reported previously. [11 12]
**Hearts with an univentricular atrioventricular connection**	1. Double inlet left atrioventricular connection;<br>2. Absence of one atrioventricular connection: non HLHS mitral atresia and tricuspid atresia; [13 14]
**Unbalanced atrioventricular septal defect**	A common atrioventricular junction and valve with only one completely well-developed ventricle (AVSD), but 75% or more commitment to the dominant ventricle, and a f-SV management pathway.
**Atrial isomerism with f-SV**	Atrial isomerism with only one well developed ventricle and a f-SV management pathway, irrespective of type of atrioventricular connection.
**Other major primary congenital heart diagnoses with f-SV circulation**	CHDs where due to the presence of a hypoplastic ventricle or a straddling atrioventricular valve. The management pathway entailed staged stage one procedures for f-SV. [15]

Table S2. Procedures on the established pathway of palliation for functionally single ventricle (f-SV) disease.

**Pre-pathway**
Procedures that occurred after the child's birth (foetal procedures were not included) and before the first stage of palliative surgery.[12 14]
**Palliative first stage procedures**
Procedures that are routinely the first surgical palliation including: [3 15]<br>Type A) Norwood type operations inclusive of Norwood with Modified R Blalock interposition shunt and with right ventricle to pulmonary artery valveless conduit (Sano) and other Damus-Kaye-Stansel type procedures.<br>Type B) All types of coarctation and interrupted arch repairs, with or without pulmonary arterial banding.<br>Type C) HLHS hybrid procedures, involving bilateral pulmonary arterial bands and ductal stenting, either at the same procedure or separated by up to 2 weeks as defined by NCHDA.<br>Type D) procedures to secure pulmonary blood flow, including all types of systemic-to-pulmonary arterial shunts, stent placement in arterial duct (PDA), pulmonary valvotomy and other operations to relieve right ventricular outflow tract obstruction, and cardiac catheterisations to perforate or widen the pulmonary valve.<br>Type E) procedures to protect the pulmonary vascular bed from excessive flow, (e.g.: pulmonary trunk or arterial bands).
**Stage two procedures**
Glenn - Construction of a bidirectional superior cavopulmonary (Glenn) anastomosis (BCPA), including when BCPA procedures were contemporaneous with other procedures that are sometimes required at the same time (e.g. pulmonary arterial reconstruction).<br>Comprehensive stage two which is a combination of aorto-pulmonary amalgamation and augmentation with construction of a BCPA.
**Stage three procedure**
Total cavopulmonary connection procedures (TCPC or Fontan). This included Fontan-type procedures when they occurred contemporaneously with other procedures that are sometimes clinically required at the same time (e.g.: atrioventricular valve repair).

BCPA: bidirectional cavopulmonary anastomosis; f-SV=functionally single ventricle; HLHS=hypoplastic left heart syndrome; TCPC: Total cavopulmonary connection.

Table S3. Concurrent procedures that may occur alongside the established pathway for f-SV disease.

**The following are concurrent procedures for stage one, two and three:**
Pulmonary venous anomaly repair
Totally anomalous pulmonary venous connection repair.
Partially anomalous pulmonary venous connection repair.
Pulmonary vein stenosis repair.
Pulmonary vein procedure.
Atrioventricular valve repairs
Tricuspid valvar procedure.
Tricuspid leaflet (valvoplasty) procedure.
Tricuspid valvar annuloplasty.
Mitral leaflet (valvoplasty) procedure.
Mitral valvar annuloplasty.
Common atrioventricular valvar leaflet (valvoplasty) procedure.
Atrioventricular septal defect (AVSD): suturing together superior + inferior bridging leaflets to left ventricular side of septum ('cleft').
Atrioventricular valvar procedure in double inlet ventricle.
Tricuspid valvar replacement.
Tricuspid valve repair converted to tricuspid valvar replacement.
Mitral valvar procedure.
Mitral valvar replacement.
Mitral valve repair converted to mitral valvar replacement.
Atrioventricular septal defect procedure.
Atrioventricular septal defect (AVSD): right atrioventricular valvar procedure.
Atrioventricular septal defect (AVSD): left atrioventricular valvar procedure.
Common atrioventricular valve replacement.
Common atrioventricular valve repair converted to atrioventricular valvar replacement.
Atrioventricular septal defect (AVSD): left atrioventricular valvar replacement.
Atrioventricular septal defect (AVSD): complete (common valve orifice) repair.
Atrioventricular valvar repair.
Pulmonary artery plasty
Pulmonary arterioplasty/ reconstruction.
Pulmonary arterioplasty/ reconstruction: central (proximal to hilar bifurcation).
Pulmonary arterioplasty/ reconstruction: peripheral (at-beyond hilar bifurcation).
Procedure involving pulmonary artery.
Pacemaker. epicardial
Pacemaker system placement: permanent epicardial.
Pacemaker system placement: permanent.
Pacemaker procedure.
**The following are concurrent procedures for stage two and three only:**
Damus redo / revision of systemic outflow
Damus-Kaye-Stansel type procedure: pulmonary trunk to aorta end/side anastomosis.
Subaortic obstruction relief
Subaortic muscular shelf obstruction resection

VSD enlargement
Right ventricular outflow tract procedure.
Supravalvar aortic stenosis repair.
Arch repair
Coarctation-hypoplasia of aorta repair.
Aortic coarctation-hypoplasia repair by resection & end to end anastomosis.
Aortic coarctation-hypoplasia repair by patch aortoplasty.
Aortic coarctation-hypoplasia repair by subclavian flap aortoplasty.
Aortic arch repair.
**The following are concurrent procedures for stage three only:**
Maze operation
Maze operation.

Table S4. Assignment of additional off pathway procedures into groups.

Output of activity algorithm - detailed group	Procedure Grouping
Bypass cardiac procedures	Surgical and hybrid procedure (Surgery)
Non-bypass cardiac procedures	Surgical and hybrid procedure (Surgery)
Hybrid involving surgery and catheter procedures	Surgical and hybrid procedure (Surgery)
Implantable cardioverter defibrillator (non-surgical)	Procedural catheters and Electrophysiology (Catheter intervention)
Pacemaker (non-surgical)	Procedural catheters and Electrophysiology (Catheter intervention)
Electrophysiological study (non-surgical)	Procedural catheters and Electrophysiology (Catheter intervention)

Non-contributory procedures were removed from the analysis, i.e., diagnostic catheters, extracorporeal life support procedures.

Additional procedures were grouped based on an algorithm first introduced/applied in the annual report of The National Institute for Cardiovascular Outcomes Research (NICOR): "National congenital heart disease audit 2014-17 summary report", https://web.nicor.org.uk/CHD/an_paeds.nsf/9791867eff401e0d8025716f004bb8f2/5983f27e0b3ff3b080257d5d005cec4a/$FILE/Congenital%20Summary%20Report%202014-17.pdf.

Table S5. Frequency of additional procedures or off-pathway in the first year of life for patients with functionally single ventricle (f-SV) disease by diagnosis subtype.

**Top 3 most common additional surgeries and catheter interventions in 701 HLHS patients with transplant-free survival beyond one-year of age**		
surgery	Modified right Blalock interposition shunt.	22 (3.1%)
	Norwood type procedure.	21 (3%)
	Atrial septectomy.	21 (3%)
Catheter intervention	Balloon dilation of aortic recoarctation.	107 (15.3%)
	Stent placement in cardiac conduit.	48 (6.8%)
	Balloon dilation of left pulmonary artery.	28 (4%)
**Top 3 most common additional surgeries and catheter interventions in 565 HLHS patients with transplant-free survival less than one-year since birth**		
surgery	Norwood type procedure.	25 (4.4%)
	Procedure involving constructed cardiac conduit-shunt.	17 (3%)
	Modified right Blalock interposition shunt.	15 (2.7%)
Catheter interventi	Balloon atrial septostomy by pull back (Rashkind).	21 (3.7%)
	Balloon dilation of aortic recoarctation.	17 (3%)
	Balloon dilation of left pulmonary artery.	8 (1.4%)
**Top 3 most common additional surgeries and catheter interventions in 195 f-SV with atrial isomerism patients with transplant-free survival beyond one-year of age**		
surgery	Modified left Blalock interposition shunt.	7 (3.6%)
	Pulmonary trunk band (PA band).	6 (3.1%)
	Central systemic-to-pulmonary arterial interposition shunt.	5 (2.6%)
Catheter intervention	Balloon dilation of left pulmonary artery.	5 (2.6%)
	Balloon dilation of right pulmonary artery.	4 (2.1%)
	Balloon dilation of aortic recoarctation.	3 (1.5%)
**Top 3 most common additional surgeries and catheter interventions in 48 f-SV with atrial isomerism patients with transplant-free survival less than one-year since birth**		
surgery	Pulmonary vein stenosis repair.	4 (8.3%)
	Modified right Blalock interposition shunt.	3 (6.3%)
	Pulmonary arterioplasty/ reconstruction: central (proximal to hilar bifurcation).	2 (4.2%)
Catheter intervention	Balloon dilation of left pulmonary artery.	2 (4.2%)
	Stent placement in cardiac conduit.	2 (4.2%)
	Balloon dilation of pulmonary vein or pathway.	2 (4.2%)
**Top 3 most common additional surgeries and catheter interventions in 289 double inlet left ventricle patients with transplant-free survival beyond one-year of age.**		
surgery	Modified right Blalock interposition shunt.	11 (3.8%)
	Pulmonary trunk band (PA band).	9 (3.1%)
	Pulmonary arterioplasty/ reconstruction: central (proximal to hilar bifurcation).	6 (2.1%)

Catheter intervention	Balloon atrial septostomy by pull back (Rashkind).	12 (4.2%)
	Balloon dilation of aortic recoarctation.	11 (3.8%)
	Systemic-to-pulmonary collateral artery(ies) (MAPCA(s)) occlusion.	2 (0.7%)
**Top 3 most common additional surgeries and catheter interventions in 39 double inlet left ventricle patients with transplant-free survival less than one-year since birth**		
surgery	Pulmonary trunk band (PA band).	3 (7.7%)
	Modified right Blalock interposition shunt.	2 (5.1%)
	Procedure involving constructed cardiac conduit-shunt.	2 (5.1%)
Catheter intervention	Balloon dilation of aortic recoarctation.	2 (5.1%)
	Stent placement in left pulmonary artery.	2 (5.1%)
	Balloon dilation of left pulmonary artery.	1 (2.6%)
**Top 3 most common additional surgeries and catheter interventions in 362 tricuspid atresia patients with transplant-free survival beyond one-year of age.**		
surgery	Modified right Blalock interposition shunt.	15 (4.1%)
	Modified left Blalock interposition shunt.	13 (3.6%)
	Atrial septectomy.	12 (3.3%)
Catheter intervention	Balloon atrial septostomy by pull back (Rashkind).	20 (5.5%)
	Balloon dilation of aortic recoarctation.	9 (2.5%)
	Balloon dilation of left pulmonary artery.	6 (1.7%)
**Top 3 most common additional surgeries and catheter interventions in 86 tricuspid atresia patients with transplant-free survival less than one-year since birth**		
surgery	Modified right Blalock interposition shunt.	7 (8.1%)
	Pulmonary arterioplasty/ reconstruction: central (proximal to hilar bifurcation).	3 (3.5%)
	Pulmonary trunk band (PA band).	3 (3.5%)
Catheter intervention	Balloon atrial septostomy by pull back (Rashkind).	3 (3.5%)
	Procedure involving constructed cardiac conduit-shunt.	2 (2.3%)
	Transluminal retrieval of device or foreign body.	1 (1.2%)
**Top 3 most common additional surgeries and catheter interventions in 90 mitral atresia without HLHS patients with transplant-free survival beyond one-year of age.**		
surgery	Atrial septectomy.	9 (10%)
	Modified right Blalock interposition shunt.	3 (3.3%)
	Pulmonary trunk band (PA band).	3 (3.3%)
Catheter intervention	Balloon atrial septostomy by pull back (Rashkind).	5 (5.6%)
	Stent placement in left pulmonary artery.	3 (3.3%)
	Transluminal occlusion of systemic-to-pulmonary collateral artery(ies) (MAPCA(s)) with coil-device.	3 (3.3%)

**Top 3 most common additional surgeries and catheter interventions in 22 mitral atresia without HLHS patients with transplant-free survival less than one-year since birth**		
surgery	Atrial septectomy.	1 (4.5%)
	Pulmonary trunk band (PA band).	1 (4.5%)
	Patent arterial duct (PDA) closure: surgical.	1 (4.5%)
Catheter intervention	Balloon dilation of systemic vein or pathway.	1 (4.5%)
	Balloon dilation of left pulmonary artery.	1 (4.5%)
	Stent placement in right pulmonary artery.	1 (4.5%)
**Top 3 most common additional surgeries and catheter interventions in 147 unbalanced AVSD patients with transplant-free survival beyond one-year of age.**		
surgery	Pulmonary trunk band (PA band).	5 (3.4%)
	Totally anomalous pulmonary venous connection repair.	3 (2%)
	Open adjustment of pulmonary trunk band.	2 (1.4%)
Catheter intervention	Balloon dilation of aortic recoarctation.	7 (4.8%)
	Stent placement in left pulmonary artery.	5 (3.4%)
	Stent placement in arterial duct (PDA).	3 (2%)
**Top 3 most common additional surgeries and catheter interventions in 84 unbalanced AVSD patients with transplant-free survival less than one-year since birth**		
surgery	Pulmonary trunk band (PA band).	5 (6%)
	Modified right Blalock interposition shunt.	5 (6%)
	Takedown of Glenn.	4 (4.8%)
Catheter intervention	Stent placement in cardiac conduit.	2 (2.4%)
	Cardiovascular catherisation occlusion procedure with coil.	2 (2.4%)
	Balloon dilation of aortic recoarctation.	2 (2.4%)
**Top 3 most common additional surgeries and catheter interventions in 130 pulmonary atresia patients with transplant-free survival beyond one-year of age.**		
surgery	Modified right Blalock interposition shunt.	16 (12.3%)
	Patent arterial duct (PDA) closure: surgical.	7 (5.4%)
	Modified Blalock interposition shunt.	6 (4.6%)
Catheter intervention	Balloon atrial septostomy by pull back (Rashkind).	17 (13.1%)
	Balloon dilation of pulmonary valve.	15 (11.5%)
	Stent placement in arterial duct (PDA).	6 (4.6%)
**Top 3 most common additional surgeries and catheter interventions in 8 pulmonary atresia patients with transplant-free survival less than one-year since birth**		
surgery	Central systemic-to-pulmonary arterial interposition shunt.	1 (12.5%)
	Procedure involving constructed cardiac conduit-shunt.	1 (12.5%)
	-	-
Ca th	Balloon atrial septostomy by pull back (Rashkind).	1 (12.5%)

	-	-
	-	-
**Top 3 most common additional surgeries and catheter interventions in 484 other f-SV patients with transplant-free survival beyond one-year of age.**		
surgery	Pulmonary trunk band (PA band).	23 (4.8%)
	Modified right Blalock interposition shunt.	21 (4.3%)
	Atrial septectomy.	17 (3.5%)
Catheter intervention	Balloon atrial septostomy by pull back (Rashkind).	18 (3.7%)
	Balloon dilation of aortic recoarctation.	13 (2.7%)
	Procedure involving constructed cardiac conduit-shunt.	8 (1.7%)
**Top 3 most common additional surgeries and catheter interventions in 97 other f-SV patients with transplant-free survival less than one-year since birth**		
surgery	Pulmonary arterioplasty/ reconstruction.	4 (7%)
	Procedure involving constructed cardiac conduit-shunt.	3 (5.3%)
	Central systemic-to-pulmonary arterial interposition shunt.	3 (5.3%)
Catheter interventi	Balloon dilation of left pulmonary artery.	3 (5.3%)
	Stent placement in cardiac conduit.	1 (1.8%)
	Venovenous collateral occlusion with device.	1 (1.8%)

Data are n(%). Procedures may arise more than once in the same patient.

Table S6. Number of additional interventions in functionally univentricular hearts (f-SV) patients during the first year of their lives.

	**Patients with transplant-free survival less than one year since birth (n=909)**	**Patients with transplant-free survival beyond one year of age (n=2,398)**
**Number of additional interventions in the first year of life**		
No additional procedure	586 (64.5%)	1,642 (68.5%)
Only one additional surgery	129 (14.2%)	211 (8.8%)
Only one additional catheter intervention	72 (7.9%)	293 (12.2%)
Multiple additional interventions (any type)	122 (13.4%)	252 (10.5%)
**Number of additional surgeries in the first year of life**		
No additional surgery	674 (74.1%)	1,999 (83.4%)
One additional surgery	179 (19.7%)	316 (13.2%)
Multiple additional surgeries	56 (6.2%)	83 (3.5%)
**Number of additional catheter interventions in the first year of life**		
No additional catheter	749 (82.4%)	1,897 (79.1%)
Only one additional catheter intervention	120 (13.2%)	391 (16.3%)
Multiple additional catheter interventions	40 (4.4%)	110 (4.6%)

Non-contributory procedures were removed from the analysis, i.e., diagnostic catheters, extracorporeal life support procedures.

Data are n(%). Non-contributory procedures were removed from the analysis, i.e., diagnostic catheters, extracorporeal life support procedures.

Table S7. Unadjusted hazard ratios and subdistribution hazard ratios (with 95% confidence intervals) for survival and Fontan completion amongst children with transplant-free survival beyond one-year of age.

Risk factors	Survival outcome beyond age one-year	Fontan completion
	HR (95%CI)	SHR (95%CI)
**Number of additional surgeries in the first year of life (Ref: no additional surgery)**		
1	1.20 (0.85-1.70)	0.95 (0.73-1.24)
>=2	3.55 (1.81-6.95)***	0.68 (0.40-1.16)
**Number of additional interventional catheters in the first year of life (Ref: no additional catheter intervention)**		
1	0.84 (0.54-1.31)	1.13 (0.97-1.33)
>=2	2.35 (1.60-3.47)***	0.78 (0.55-1.10)
**f-SV subtypes (Ref: HLHS)**		
f-SV with atrial isomerism	1.46 (1.00-2.14)	0.16 (0.07-0.33)***
f-SV with atrial isomerism × follow-up time	-	1.30 (1.18-1.44)***
Double inlet left ventricle	0.41 (0.20-0.85)*	0.82 (0.59-1.13)
Tricuspid atresia	0.33 (0.20-0.54)***	0.27 (0.14-0.49)***
Tricuspid atresia × follow-up time	-	1.36 (1.27-1.47)***
Mitral atresia without HLHS	0.44 (0.23-0.83)	0.28 (0.13-0.59)**
Mitral atresia without HLHS × follow-up time	-	1.30 (1.13-1.49)***
Unbalanced atrioventricular septal defect (AVSD)	1.77 (1.18-2.66)**	0.54 (0.41-0.71)***
Pulmonary Atresia without other complex features but with f-SV	0.43 (0.20-0.93)*	0.69 (0.56-0.87)**
Other f-SV types	0.61 (0.46-0.81)**	0.27 (0.15-0.48)***
Other f-SV types × follow-up time	-	1.23 (1.14-1.32)***
**Additional cardiac risk factor (Ref: none)**	2.43 (1.68-3.53)	0.59 (0.47-0.75)***
Additional cardiac risk factor × follow-up time	1.09 (1.02-1.17)*	-
**Surgical variables**		
Pre-stage one procedure (any type) (Ref: none)	0.88 (0.60-1.29)	0.92 (0.74-1.15)
Stage one with concurrent surgery (Ref: none)	1.14 (0.68-1.90)	0.78 (0.59-1.04)
No stage two procedure (Ref: Glenn)	14.21 (8.51-23.71)***	0.33 (0.23-0.49)***
No stage two procedure x follow-up time	0.62 (0.51-0.76)***	-
Comprehensive stage two (Ref: Glenn)	1.20 (0.82-1.75)	1.07 (0.87-1.31)
Stage two with concurrent surgery (Ref: none)	0.74 (0.50-1.11)	1.04 (0.94-1.15)
Stage two with concurrent surgery x follow-up time	1.11 (1.03-1.19)**	
**Non cardiac variables**		
Male (Ref: female)	0.83 (0.66-1.04)	1.18 (1.06-1.31)**
Prematurity (Ref: Born >=37 weeks gestation)	0.81 (0.41-1.60)	0.90 (0.71-1.12)
Congenital non-cardiac comorbidity (Ref: none)	1.53 (1.21-1.92)***	0.50 (0.39-0.65)***
Congenital non-cardiac comorbidity × follow-up time		1.09 (1.04-1.14)***

Low weight at first procedure (Ref: >=2.5kg)	1.63 (1.19-2.24)**	0.94 (0.81-1.09)
Acquired comorbidity at first procedure (Ref: none)	1.38 (0.89-2.16)	0.70 (0.40-1.22)
Increased severity of illness at first procedure (Ref: none)	1.40 (0.78-2.51)	0.92 (0.69-1.23)
Age (years) at first procedure	0.95 (0.83-1.10)	0.58 (0.50-0.67)***
Age (years) at first procedure × follow-up time	-	1.05 (1.04-1.07)***
Born after April 2009 (Ref: born before April 2009)	0.87 (0.58-1.31)	1.08 (0.93-1.25)

Patients with transplant-free survival less than one-year since birth (died, had heart transplant or censored before the age of one) were not included in the model. Centre-clustered standard errors computed.

HR = hazard ratio; SHR = subdistribution hazard ratio.

Significance level (p-value): 0.05 * 0.01 ** 0.001 ***

Interpretation of coefficients for covariates with time-varying interaction term. Consider A as a categorical covariate and the hazard regression coefficients for A and the time interaction term A × follow-up time (years from birth) are expressed as β and γ, respectively. The estimation of $\beta+\gamma*$follow-up time represents the change in the expected log of the hazard ratio relative to the reference. In the table, we report the baseline hazard ratio and time changing hazard ratio by exp(β) and exp(γ). A value of exp(γ) larger than 1 indicates the mortality risk will increase with time, compared with the reference group, and vice versa.

Table S8. Breakdown of the number of additional interventions and covariates for children with transplant-free survival beyond one-year of age.

**Additional surgeries in the first year of life**					
	**Patient number**	**Number (%) of additional surgeries in the first year of life**			
		**None**	**1**	**>=2**	
**Total**	2398	1999 (83.4%)	316 (13.2%)	83 (3.5%)	
**Breakdown by covariates**					**p-value***
**f-SV subtypes**					0.49
Hypoplastic left heart syndrome (HLHS)	701	575 (82.0%)	99 (14.1%)	27 (3.9%)	
f-SV with atrial isomerism	195	166 (85.1%)	26 (13.3%)	3 (1.5%)	
Double inlet left ventricle	289	253 (87.5%)	26 (9.0%)	10 (3.5%)	
Tricuspid atresia	362	302 (83.4%)	47 (13.0%)	13 (3.6%)	
Mitral atresia without HLHS	90	74 (82.2%)	15 (16.7%)	1 (1.1%)	
Unbalanced atrioventricular septal defect (AVSD)	147	132 (89.8%)	13 (8.8%)	2 (1.4%)	
Pulmonary Atresia without other complex features but with f-SV	130	91 (70.0%)	27 (20.8%)	12 (9.2%)	
Other f-SV types	484	406 (83.9%)	63 (13.0%)	15 (3.1%)	
**Additional cardiac risk factor**	165	123 (74.5%)	29 (17.6%)	13 (7.9%)	<0.001
**Pre-stage one procedure (any type)**	246	214 (87.0%)	22 (8.9%)	10 (4.1%)	0.08
**Stage one with concurrent surgery**	100	87 (87.0%)	10 (10.0%)	3 (3.0%)	0.67
**Stage two subtypes**					<0.001
No stage two procedure	121	87 (71.9%)	26 (21.5%)	8 (6.6%)	
Glenn	2127	1792 (84.3%)	270 (12.7%)	65 (3.1%)	
Comprehensive stage two	150	120 (80.0%)	20 (13.3%)	10 (6.7%)	
**Stage two with concurrent surgery**	875	729 (83.3%)	112 (12.8%)	34 (3.9%)	0.64
**Male**	1411	1168 (82.8%)	193 (13.7%)	50 (3.5%)	0.66
**Prematurity**	140	101 (72.1%)	28 (20.0%)	11 (7.9%)	<0.001
**Congenital non-cardiac comorbidity**	397	320 (80.6%)	56 (14.1%)	21 (5.3%)	0.07

**Low weight baby (below 2.5Kg) at first procedure**	197	134 (68.0%)	50 (25.4%)	13 (6.6%)	<0.001
**Acquired comorbidity at first procedure**	91	72 (79.1%)	14 (15.4%)	5 (5.5%)	0.32
**Increased severity of illness at first procedure**	229	164 (71.6%)	45 (19.7%)	20 (8.7%)	<0.001
**Recent data - Born after April 2009**	1203	970 (80.6%)	178 (14.8%)	55 (4.6%)	<0.001
**Age (days) at first procedure median (IQR)**	2398	8 (5-38)	8 (5-38)	8 (4-30)	-
**Additional interventional catheters in the first year of life**					
	**Patient number**	**Number (%) of additional catheter interventions in the first year of life**			
		**None**	**1**	**>=2**	
**Total**	2398	1897 (79.1%)	391 (16.3%)	110 (4.6%)	
**Breakdown by covariates**					**p-value***
**f-SV subtypes**					<0.001
Hypoplastic left heart syndrome (HLHS)	701	457 (65.2%)	180 (25.7%)	64 (9.1%)	
f-SV with atrial isomerism	195	176 (90.3%)	13 (6.7%)	6 (3.1%)	
Double inlet left ventricle	289	250 (86.5%)	33 (11.4%)	6 (2.1%)	
Tricuspid atresia	362	313 (86.5%)	40 (11.0%)	9 (2.5%)	
Mitral atresia without HLHS	90	74 (82.2%)	11 (12.2%)	5 (5.6%)	
Unbalanced atrioventricular septal defect (AVSD)	147	123 (83.7%)	20 (13.6%)	4 (2.7%)	
Pulmonary Atresia without other complex features but with f-SV	130	84 (64.6%)	40 (30.8%)	6 (4.6%)	
Other f-SV types	484	420 (86.8%)	54 (11.2%)	10 (2.1%)	
**Additional cardiac risk factor**	165	119 (72.1%)	33 (20.0%)	13 (7.9%)	0.04
**Pre-stage one procedure (any type)**	246	213 (86.6%)	27 (11.0%)	6 (2.4%)	0.01
**Stage one with concurrent surgery**	100	80 (80.0%)	18 (18.0%)	2 (2.0%)	0.45
**Stage two subtypes**					<0.001
No stage two procedure	121	96 (79.3%)	17 (14.0%)	8 (6.6%)	
Glenn	2127	1699 (79.9%)	340 (16.0%)	88 (4.1%)	
Comprehensive stage two	150	102 (68.0%)	34 (22.7%)	14 (9.3%)	
**Stage two with concurrent surgery**	875	627 (71.7%)	186 (21.3%)	62 (7.1%)	<0.001
**Male**	1411	1114 (79.0%)	231 (16.4%)	66 (4.7%)	0.96

**Prematurity**	140	104 (74.3%)	29 (20.7%)	7 (5.0%)	0.28
**Congenital non-cardiac comorbidity**	397	301 (75.8%)	76 (19.1%)	20 (5.0%)	0.18
**Low weight baby (below 2.5Kg) at first procedure**	197	136 (69.0%)	48 (24.4%)	13 (6.6%)	<0.001
**Acquired comorbidity at first procedure**	91	66 (72.5%)	15 (16.5%)	10 (11.0%)	0.02
**Increased severity of illness at first procedure**	229	144 (62.9%)	66 (28.8%)	19 (8.3%)	<0.001
**Recent data - Born after April 2009**	1203	887 (73.7%)	234 (19.5%)	82 (6.8%)	<0.001
**Age (days) at first procedure median (IQR)**	2398	8 (4-41)	8 (4-56)	9 (5-24)	-

Data are n (%) or median value (IQR).

* Fisher's exact test between the number of additional surgeries during the first year of patients' life and categorical covariates.

Low weight includes imputed data, 56 (2.3%).

Figure S1. Inclusions and exclusions.

The process of case ascertainment of the study cohort of 3,307 patients with functionally univentricular hearts (f-SV) disease from the National Congenital Heart Diseases Audit (NCHDA) data set with specific exclusions stated at each step.

Inclusion criteria of HLHS see in the reference; [11 12] inclusion of other major CHD diagnosis with f-SV circulation patients see in the reference. [13 14 15]

AVSD=atrioventricular septal defect; f-SV=functionally single ventricle; HLHS=hypoplastic left heart syndrome.

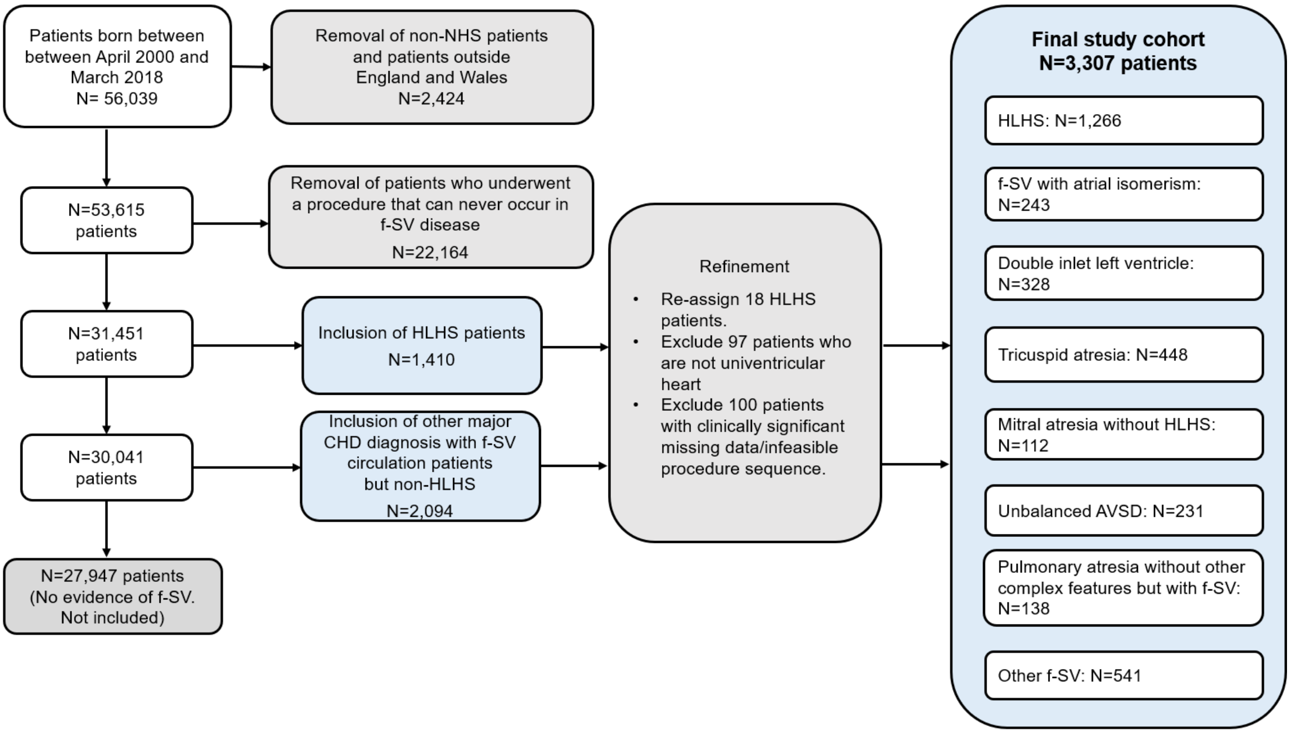

Figure S2. Planned treatment pathways and outcomes for 909 patients with functionally single ventricle (f-SV) who exhibited a transplant-free survival less than an one year since birth.

The subtype of staged procedure is presented as n(%), where the ratio is computed based on the number of patients who had stage one, two or three procedure.

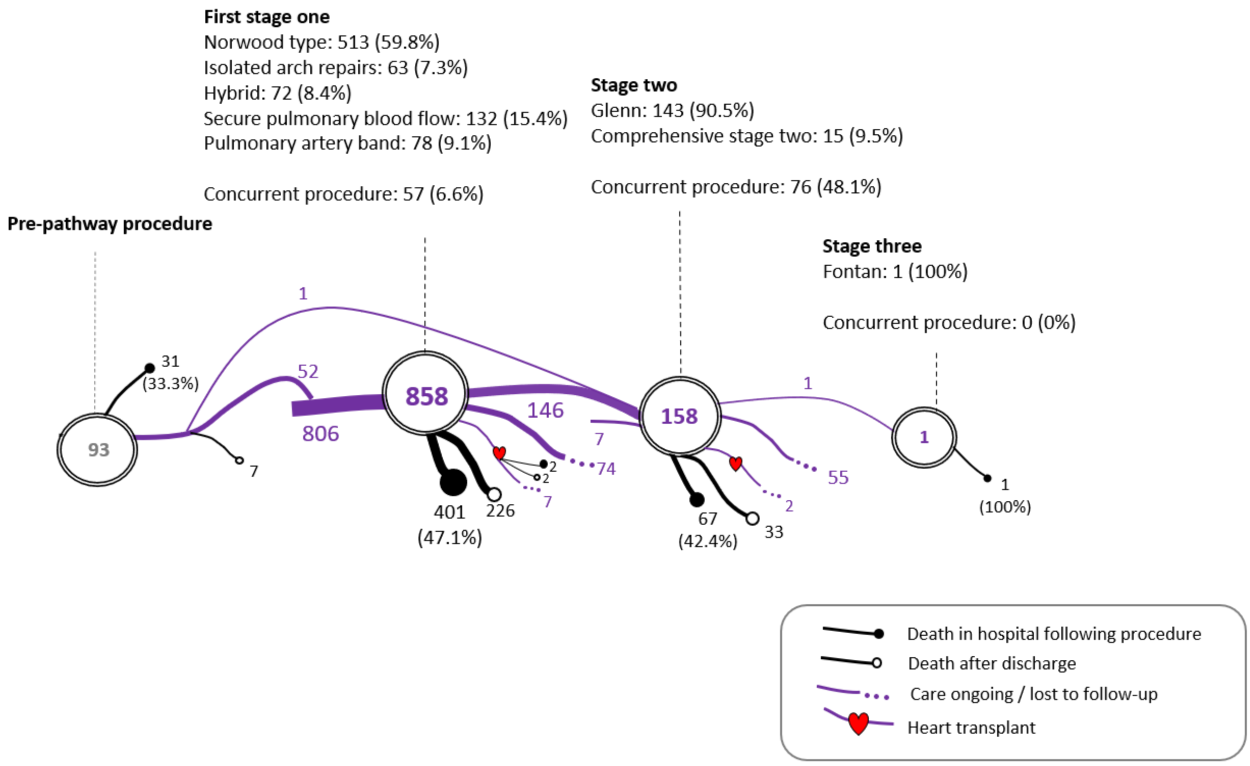

Figure S3. Percentage of case mix in functionally single ventricle (f-SV) patients by birth year.

This figure is the supplementary Figure S3 from Brown KL, Huang Q, Hadjicosta E, Seale AN, Tsang V, Anderson D, Barron D, Bellsham-Revell H, Pagel C, Crowe S, et al. Long-term survival and center volume for functionally single-ventricle congenital heart disease in England and Wales. The Journal of Thoracic and Cardiovascular Surgery. 2023;166:306-16

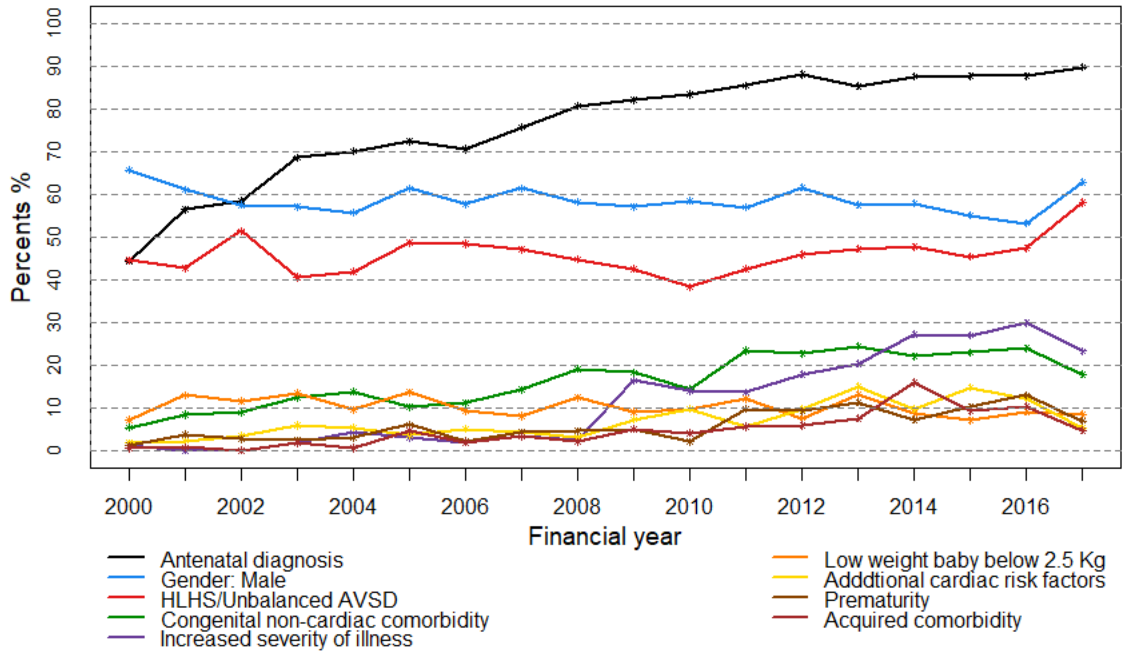

Figure S4. Percentage of stage one subtype performed in functionally single ventricle (f-SV) patients by birth year.

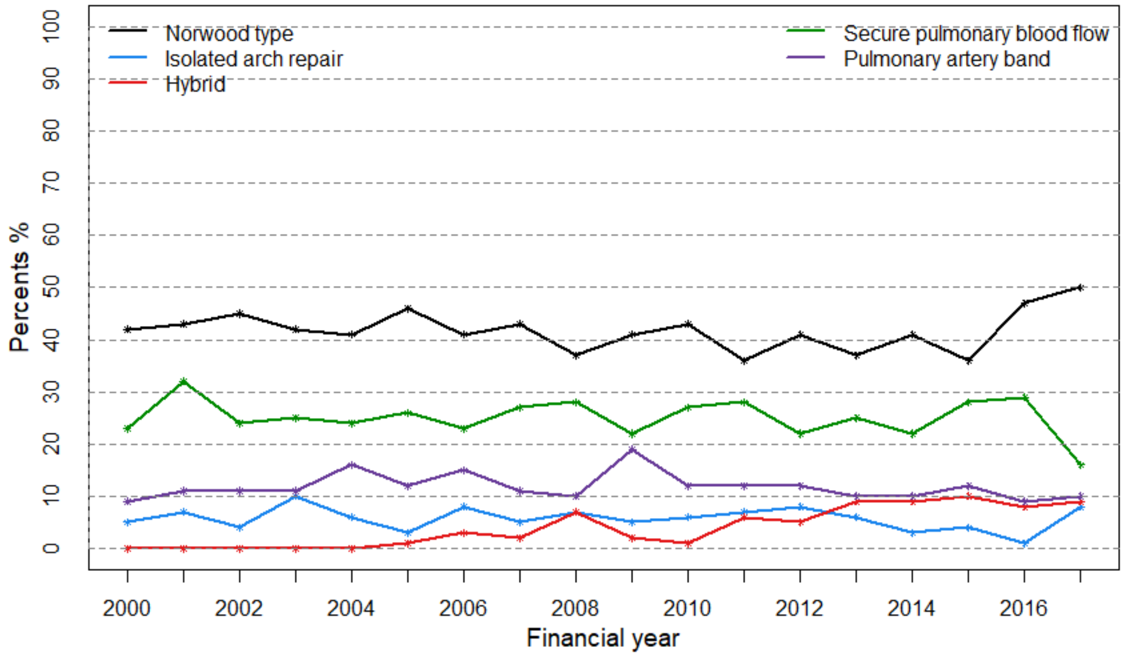

Figure S5. One-year and 5-year survival rates with 95% CI by birth year for functionally single ventricle (f-SV) patients.

This figure is the supplementary Figure S2 from Brown KL, Huang Q, Hadjicosta E, Seale AN, Tsang V, Anderson D, Barron D, Bellsham-Revell H, Pagel C, Crowe S, et al. Long-term survival and center volume for functionally single-ventricle congenital heart disease in England and Wales. The Journal of Thoracic and Cardiovascular Surgery. 2023;166:306-16.

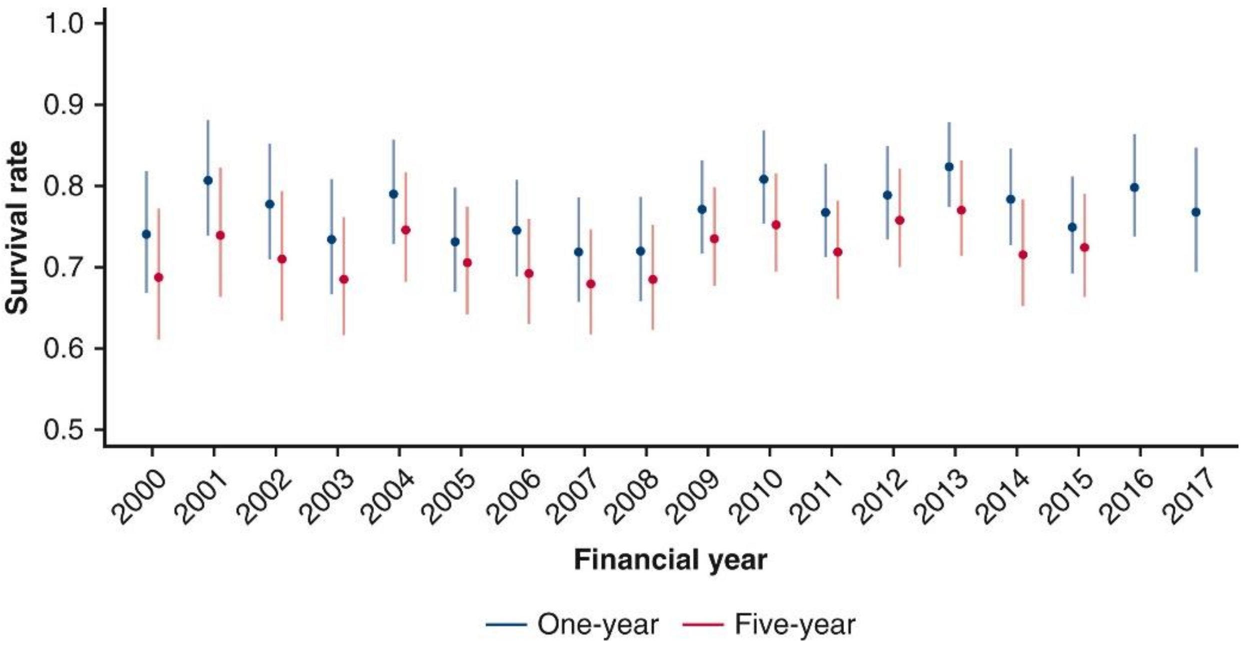

Supplement: Supplementary file 1 — Tables S1–S8 Figures S1–S5 [file JAH3-13-e033068-s001.pdf]
